# Olipudase alfa for treatment of acid sphingomyelinase deficiency (ASMD): safety and efficacy in adults treated for 30 months

**DOI:** 10.1007/s10545-017-0123-6

**Published:** 2018-01-05

**Authors:** Melissa P. Wasserstein, George A. Diaz, Robin H. Lachmann, Marie-Hélène Jouvin, Indrani Nandy, Allena J. Ji, Ana Cristina Puga

**Affiliations:** 1 Present Address: Children’s Hospital at Montefiore, Albert Einstein College of Medicine, Bronx, NY USA; 2 Genetics and Genomics Sciences, Icahn School of Medicine at Mount Sinai, New York, NY USA; 3 National Hospital for Neurology and Neurosurgery, London, UK; 4 Present Address: Agios Pharmaceuticals, Cambridge, MA USA; 5 Clinical Sciences and Operations, Sanofi Genzyme, Cambridge, MA USA; 6 Biomarkers and Clinical Bioanalyses, Sanofi Genzyme, Framingham, MA USA; 7 Clinical Development, Rare Diseases, Sanofi Genzyme, 1, Avenue Pierre Brossolette, 91385 Chilly-Mazarin, France

## Abstract

Olipudase alfa, a recombinant human acid sphingomyelinase (ASM), is an enzyme replacement therapy for the treatment of nonneurologic manifestations of acid sphingomyelinase deficiency (ASMD). This ongoing, open-label, long-term study (NCT02004704) assessed safety and efficacy of olipudase alfa following 30 months of treatment in five adult patients with ASMD. There were no deaths, serious or severe events, or discontinuations during 30 months of treatment. The majority of adverse events were mild and included headache, nausea, and abdominal pain. No patient developed anti-drug antibodies and there were no clinically significant adverse changes in vital signs, hematology, or cardiac safety parameters. Statistically significant reductions in liver (31%) and spleen (39%) volumes were maintained through 30 months of treatment. There was a mean increase in lung diffusing capacity of 35%, and clinically relevant improvements in infiltrative lung disease parameters. Lipid profiles improved in all patients. Improvements in bone mineral density of the spine were observed in some patients. Chitotriosidase in serum and lyso-sphingomyelin in dried blood spots decreased with olipudase alfa treatment, suggesting utility as biomarkers for monitoring treatment efficacy. Olipudase alfa is the first etiology-specific treatment in development for ASMD. This study demonstrates that treatment with olipudase alfa for 30 months is well-tolerated and associated with life-transforming sustained improvements in relevant disease clinical measures.

**Electronic supplementary material:**

The online version of this article (10.1007/s10545-017-0123-6) contains supplementary material, which is available to authorized users.

## Introduction

Acid sphingomyelinase deficiency (ASMD), an autosomal recessive lysosomal storage disorder, results from mutations in the *SMPD1* gene encoding the lysosomal enzyme acid sphingomyelinase (ASM) (Schuchman and Desnick 2017). Subsequent sphingomyelin accumulation in multiple organs causes visceral disease and neurodegeneration in severe cases. Infantile neurovisceral ASMD (historically known as Niemann-Pick disease type A [NPD A]), the most severe disease phenotype, results in death in early childhood (McGovern et al 2006). Patients with chronic visceral ASMD (NPD B) and chronic neurovisceral ASMD (NPD A/B) have onset that varies from infancy to adulthood (Wasserstein et al 2004, Wasserstein et al 2006). Morbidity from liver, lung, and hematologic disease occurs in all patients with chronic ASMD and includes hepatosplenomegaly, liver dysfunction, infiltrative lung disease, and thrombocytopenia (McGovern et al 2013, McGovern et al 2017). Growth restriction during childhood and bone disease are also common features of chronic ASMD (Wasserstein et al 2003). Pulmonary and liver disease are the main causes of death in these patients (McGovern et al 2008, Cassiman et al 2016).

Olipudase alfa, a recombinant human ASM, is an intravenous enzyme replacement therapy (ERT) for the treatment of nonneurologic ASMD manifestations (Miranda et al 2000). Gradual debulking of sphingomyelin and its catabolites via initial dose escalation was successful in a 6-month phase 1b study in five adult patients administered initial doses of 0.1 mg/kg of olipudase alfa followed by stepwise biweekly increases to reach the target dose of 3.0 mg/kg (Wasserstein et al 2015).

Olipudase alfa treatment for 6 months showed a favorable safety profile (Wasserstein et al 2015). Transient increases in ceramide, bilirubin, high-sensitivity C-reactive protein (hsCRP), and other acute phase reactants peaked 1–2 days post-dose during the dose escalation and resolved within 2 weeks. Improvements in spleen and liver volume, lung function, and lipid profiles were observed (Wasserstein et al 2015). Long-term safety and efficacy of olipudase alfa continue to be assessed in these patients, and the present analysis shows a sustained safety profile and continued improvements in multiple clinically relevant parameters following 30 months of treatment.

## Methods

### Patients and study design

This ongoing, open-label, long-term study (LTS) (NCT02004704; EudraCT Number: 2013–000051-40) follows five adult patients with chronic ASMD who previously participated in the phase 1b study (Wasserstein et al 2015). Data were analyzed for all patients after 30 months of treatment (last date 13 May 2016). The Institutional Review Board or Ethics Committee at each site approved the protocol and all patients provided written informed consent. The study was conducted according to Good Clinical Practice and in accordance with the principles of the Declaration of Helsinki.

Eligibility criteria for the phase 1b study have been previously described (Wasserstein et al 2015). Patients completing the phase 1b study with an acceptable safety profile were eligible to continue in the LTS and continued at the same olipudase alfa dose they were receiving at the end of the phase 1b study.

### Outcome measures and analyses

Safety assessments included standard hematologic and chemistry panels and continuous AE monitoring, including infusion associated reactions (IARs) as previously described (McGovern et al 2015, Wasserstein et al 2015).

Sphingomyelin and catabolite plasma ceramide were assessed by liquid chromatography-tandem mass spectrometry (LC/MS/MS). Disease biomarkers also included chitotriosidase (serum) and lyso-sphingomyelin (lyso-SPM) [dried blood spot (DBS)] determined by LC/MS/MS. Development of anti-drug antibodies was assessed as previously described (McGovern et al 2015, Wasserstein et al 2015).

Quantitative measurements of spleen and liver volumes were determined from abdominal MRI and organ volumes were expressed as multiples of normal (MN). Percent predicted, hemoglobin-adjusted, diffusing capacity of the lung for carbon monoxide (DLco) was calculated using standardized formulas (Crapo and Morris 1981, Macintyre et al 2005). High-resolution computed tomography (HRCT) assessed infiltrative lung disease. Lung field HRCT images were scored subjectively for ground glass appearance (GG), interstitial lung disease (ILD), and reticulonodular density (RD) from 0 (no disease) to 3 (severe disease) as previously described (McGovern et al 2015, Wasserstein et al 2015).

Fasting plasma lipid profiles, including measurement of total cholesterol (TC), low density lipoprotein (LDL-C), high density lipoprotein (HDL-C), and triglycerides, were measured throughout the study. Non-HDL levels were calculated post-hoc as the difference between total cholesterol and HDL-cholesterol levels (Jacobson et al 2015).

Bone marrow burden (BMB) was determined from MRIs of lumbar spine and both femurs, where image analysis enabled semiquantitative assessment of the degree of bone marrow infiltration by lipid-loaded cells (Robertson et al 2007).

Bone mineral density (BMD) was determined from dual-energy X-ray absorptiometry (DXA) bone scan images of lumbar spine and both femurs and determination of T- and Z-scores (WHO 2001). BMD was assessed using the guidance provided by the International Society for Clinical Densitometry (ISCD 2015).

Patient reported outcomes using 11 point scales from 0 (absence) to 10 (worst) included the validated Brief Fatigue Inventory (BFI) (Mendoza et al 1999) and Brief Pain Inventory-Short Form (BPI-SF) (Cleeland 2002) questionnaires to assess interference with daily activities at baseline and periodically throughout treatment.

## Statistical methods

Descriptive statistics were provided for categorical and continuous variables, change from baseline and percent change from baseline were calculated for organ volumes and DLco, and differences determined by paired t-test and the Wilcoxon-Mann-Whitney test.

## Results

### Patients and exposure

All five adult patients (three male and two female Caucasian patients) who completed the phase 1b study continued treatment in the LTS. At baseline, all patients had splenomegaly (range 7.4 to 16.1 MN), hepatomegaly (range 1.2 to 2.2 MN), impaired gas exchange (range 43 to 80% of predicted DLco), and a pro-atherogenic lipid profile. Patient characteristics have been previously published (Wasserstein et al 2015) and are summarized in Suppl. Table 1. The majority of patients (4/5) remained at the 3 mg/kg olipudase alfa target dose through 30 months of treatment. For patient 2, dose was reduced to 2 mg/kg for 6 months (months 12–18) and then to 1 mg/kg (months 18-current) due to AEs which are described below.

### Safety

There were no deaths, serious or severe events, or discontinuations during 30 months of treatment. The most common events and IARs are shown in Table 1. All patients had at least 1 AE, and almost all (826/838, 98.5%) were mild in intensity. Among 443 AEs considered related to treatment, 96 (21.7%) were considered IARs (including headache, nausea, abdominal pain, arthralgia, musculoskeletal pain, and myalgia) (Table 1). Six moderate AEs considered IARs occurred during the phase 1b study (first 6 months) and have been previously reported (Wasserstein et al, 2015). From 6 to 30 months in the LTS, five moderate AEs considered IARs included abdominal pain, hepatic pain, nausea, muscle spasm, and sensory disturbance in patient 2. There were no hypersensitivity reactions, acute phase reactions, or cytokine release syndrome. No patient developed IgG antibodies to olipudase alfa. There were no clinically significant adverse changes in vital signs, hematology, or cardiac safety parameters.

**Table 1:** Summary of adverse events

Profile of treatment emergent adverse events (TEAEs)	Patients n (%)	Events n
Any event	5 (100)	838
Mild	5 (100)	826
Moderate	2 (40)	12
Severe	0	0
Serious events, deaths, discontinuations	0	0
Treatment related events	4 (80)	443
Most common TEAEs (≥ 5% of all TEAEs)		
Headache	5 (100)	69
Nausea	5 (100)	57
Abdominal pain	5 (100)	54
Arthralgia	4 (80)	53
Fatigue	3 (60)	45
Infusion associated reactions	4 (80)	96
Mild	4 (80)	85
Moderate	2 (40)	11
Most common infusion associated reactions (≥ 5% of all IARs)		
Headache	3 (60)	23
Nausea	2 (40)	13
Abdominal pain	2 (40)	9
Arthralgia	3 (60)	7
Musculoskeletal pain	1 (20)	6
Myalgia	2 (40)	6

**Fig. 1:**
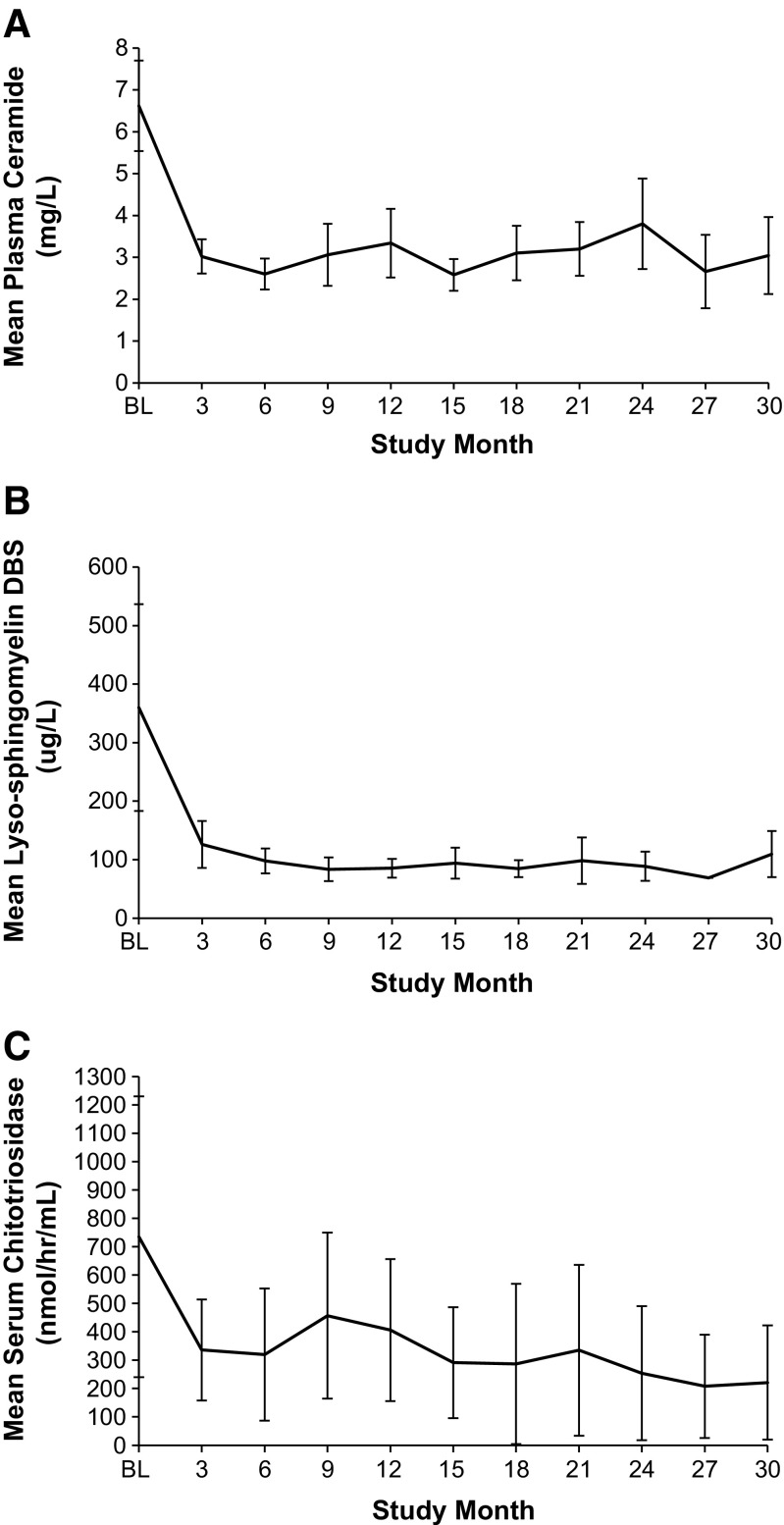
Mean plasma ceramide levels (**a**), mean lyso-sphingomyelin levels in dried blood spots (DBS) (**b**), and mean serum chitotriosidase activity (**c**) at Baseline (BL) and during treatment (30 months) with olipudase alfa. Normal range for plasma ceramide was 1.8–6.5 mg/L. The upper limit of normal for lyso-sphingomyelin in dried blood spots was <69 μg/L, and normal chitotriosidase serum levels were ≤181 nmol/h/mL (note: activity was not corrected for 2 patients heterozygous for a chitotriosidase null mutation)

Levels of inflammatory markers IL-6, IL-8, and hsCRP stable at the end of the phase 1b study (Wasserstein et al 2015) remained stable for all patients except patient 2, who had fluctuations in hsCRP (1.10 to 33.3 mg/mL; normal range 0–5) from month 6 to month 30. Plasma ceramide levels for all patients (Fig. 1a) remained within normal limits (1.8–6.5 μg/mL).

Liver function enzyme levels remained within normal ranges for all patients until month 30 when patient 4 had transient elevations in ALT (1.4× normal) and AST (2.9× normal), without corresponding AEs, and with subsequent normal levels. Total bilirubin and GGT levels remained similar to or below baseline levels for all patients. Iron levels fluctuated over time, but remained within or close to normal ranges.

During phase 1b dose escalation, patient 2 experienced IARs leading to a repeat of the 2 mg/kg dose (Wasserstein et al 2015). The patient subsequently received the target dose of 3 mg/kg through the phase 1b trial and during the first 6 months of the LTS, at which time the patient reported mild AEs 7–10 days after most infusions including nausea, headache, fatigue, achiness, intermittent abdominal pain, and occasional fever (38.3 to 40.0 °C). Episodes lasted ~3 days and were completely resolved by the next infusion. Olipudase alfa was decreased (2 mg/kg for 6 months, then to the current dose of 1 mg/kg). Decreasing the dose did not change the timing, frequency, or types of events reported.

## Efficacy

### Spleen and liver volumes

**Fig. 2:**
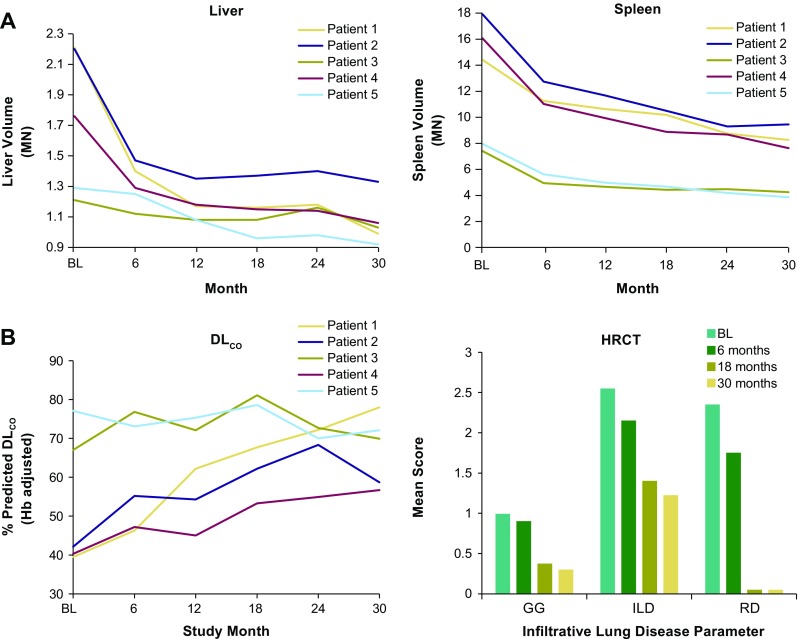
Assessment of olipudase alfa on liver and spleen volume and lung disease. (**a**) Liver and spleen volumes were calculated by integrating cross-sectional magnetic resonant images and expressed as multiples of normal (MN) where normal spleen volume (L) was assumed to be 0.2% of body weight and normal liver volume (L) to be 2.5% of body weight. (**b**) Lung disease. By-patient percent predicted DLco, adjusted for hemoglobin (Hb), at baseline and during treatment were calculated from observed values for male and female patients (Crapo and Morris 1981, Macintyre et al 2005). Degree of severity: 80% = lower limit of normal; >60%–79% = mild decrease; 40%–60% = moderate decrease; <40% = severe decrease. HRCT assessment of infiltrative lung disease at baseline and during treatment with olipudase alfa included ground glass appearance (GG), interstitial lung disease (ILD), and reticulonodular density (RD) scored on a 4 point system where 0 = No interstitial lung disease; 1 = Mild (affecting 1–25% of the lung volume); 2 = Moderate (affecting 26–50% of the lung volume); 3 = Severe (affecting 51–100% of the lung volume)

Spleen and liver volumes decreased in all patients relative to baseline (Fig. 2a). Mean spleen volumes decreased from 12.8 multiples of normal (MN) at baseline to 6.7 MN at 30 months, a 47.3% decrease from baseline (*p* < 0.0001). Mean liver volumes decreased from 1.7 MN at baseline to 1.07 MN at 30 months, a 35.6% decrease from baseline (*p* = 0.006).

### Infiltrative lung disease

Percent predicted DL_CO_ increased in all patients relative to baseline values (Fig. 2b) and improved from a mean of 53.2% (moderate) at baseline to 67.1% at 30 months (mild). The greatest changes occurred in the three patients with the lowest % predicted DL_CO_ values at baseline (<40%, in the severe range). Figure 2b also shows assessment of infiltrative lung disease with mean scores for components at baseline, 6 months, 18 months, and 30 months. The data show progressive decreases in all parameters, particularly in GG appearance and RND, which almost completely resolved.

### Fasting lipid parameters

Fasting lipid profiles are shown in Suppl. Fig. 1. By 30 months, triglycerides decreased by 42.99% (*p* = 0.02), total cholesterol by 12.7% (*p* = 0.04), LDL-C by 22.8% (*p* = 0.007), and HDL-C increased by 137.6% (*p* = 0.01). Non-HDL cholesterol level (total cholesterol minus HDL-C), was >3.37 mmol/L (>130 mg/dL) in 4/5 patients at baseline (mean 3.91 mmol/L) and was <3.37 mmol/L in all patients at 30 months (mean 2.66 mmol/L).

### Biomarker assessment

Mean lyso-SPM levels in DBS were five times above the upper limit of normal (ULN = 69 μg/L) at baseline and decreased to near-normal levels that remained stable from 6 through 30 months (Fig. 1b).

Pre-infusion serum chitotriosidase levels steadily decreased by 72.3% from 735 nmol/h/mL at baseline to 221 nmol/h/mL at 30 months (*p* = 0.0007), approaching the upper limit of normal chitotriosidase range (≤181 nmol/h/mL) (Fig. 1c). Data were not adjusted to account for two patients heterozygous for a common 24-bp duplication which reduces serum chitotriosidase activity.

### Hematology

Most patients maintained platelet counts just below normal or within the low-normal range. Patient 1 had values (57-102 × 10^9^/L) below low-normal (150 × 10^9^/L) throughout the study. Mean platelet count changes from baseline (increases) fluctuated over time [between 5.9% (month 27) and 25.7% (month 9)], and was 20.6% at 30 months. Hemoglobin levels remained similar to baseline levels (mean changes from baseline ranged from −6.1% at week 12 to 6.9% at month 24) and were within normal levels for all patients (data not shown).

### Bone density

At baseline, mean spinal T- scores were in the osteopenic range (between −1.0 and −2.5) at −1.48 ± 1.14, while Z-scores indicated normal BMD (−1.36 ± 1.26) within −1 standard deviation of the low BMD cutoff (−2.0). Both T- and Z-scores improved at 30 months (−0.94 ± 1.03 and −0.78 ± 1.11, respectively). Patient 2 (female, 32 year old at baseline) had a baseline spinal T-score (−3.06) in the osteoporotic range that improved at 18 (−2.48) and 30 months (−2.65) to values on the osteopenia/osteoporosis border. Two patients with T-scores in the osteopenic range at baseline (patient 1, male 31 years old at baseline, −1.31 and patient 4, male, 28 years old at baseline, −2.14) had scores in the normal range at 30 months (−0.76 and −0.82, respectively). Results for individual Z-scores over time were similar (not shown).

Mean femur T- and Z-scores were in the normal range at baseline (−0.38 ± 1.35 and −0.27 ± 1.46, respectively) and at 30 months (−0.28 ± 1.27 and −0.13 ± 1.4, respectively). Patient 2 had a baseline femur T-score in the osteopenic range (−2.23) and a Z-score indicating low BMD (−2.18); both improved slightly (−1.89 and −1.82, respectively) at 30 months.

### Bone marrow burden

**Fig. 3:**
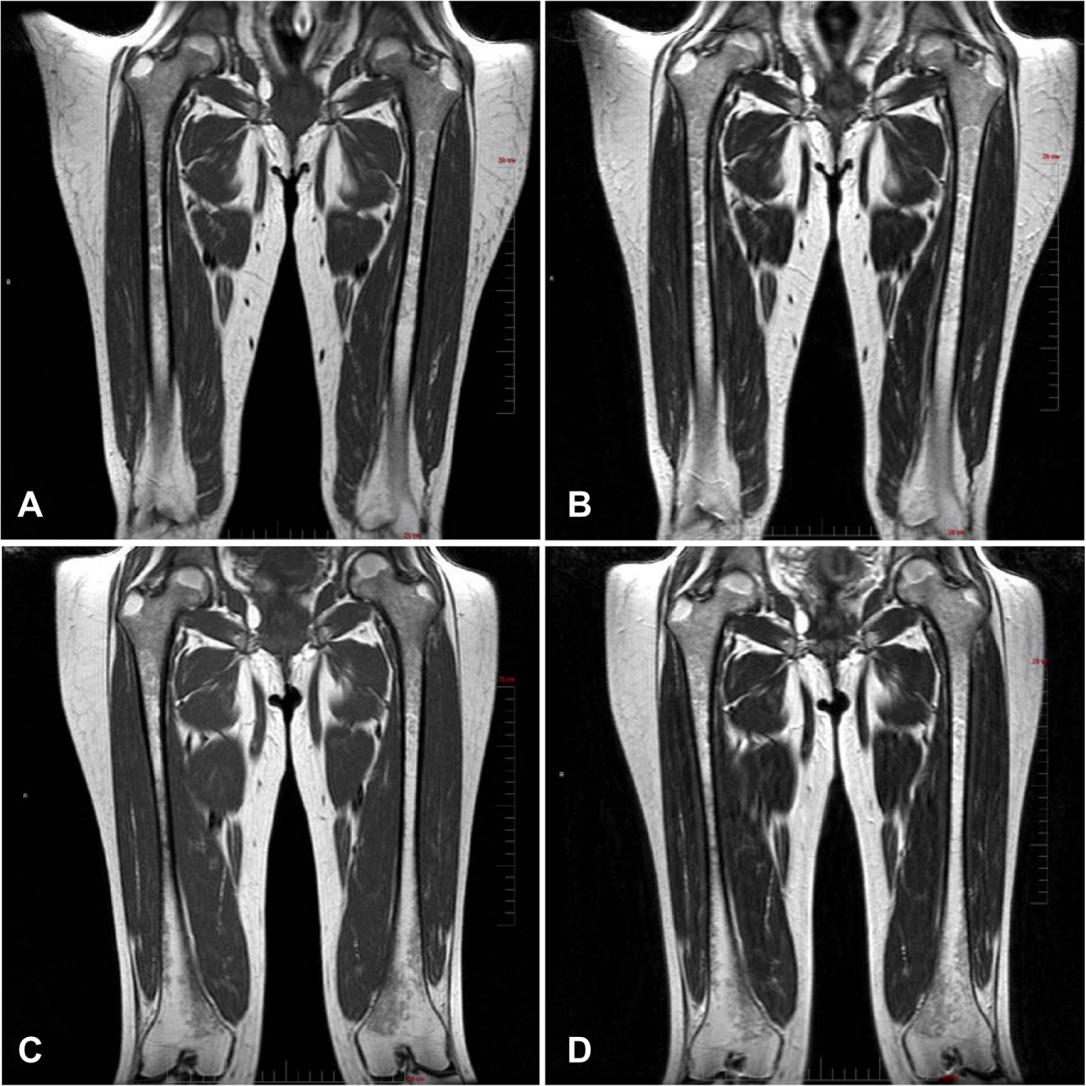

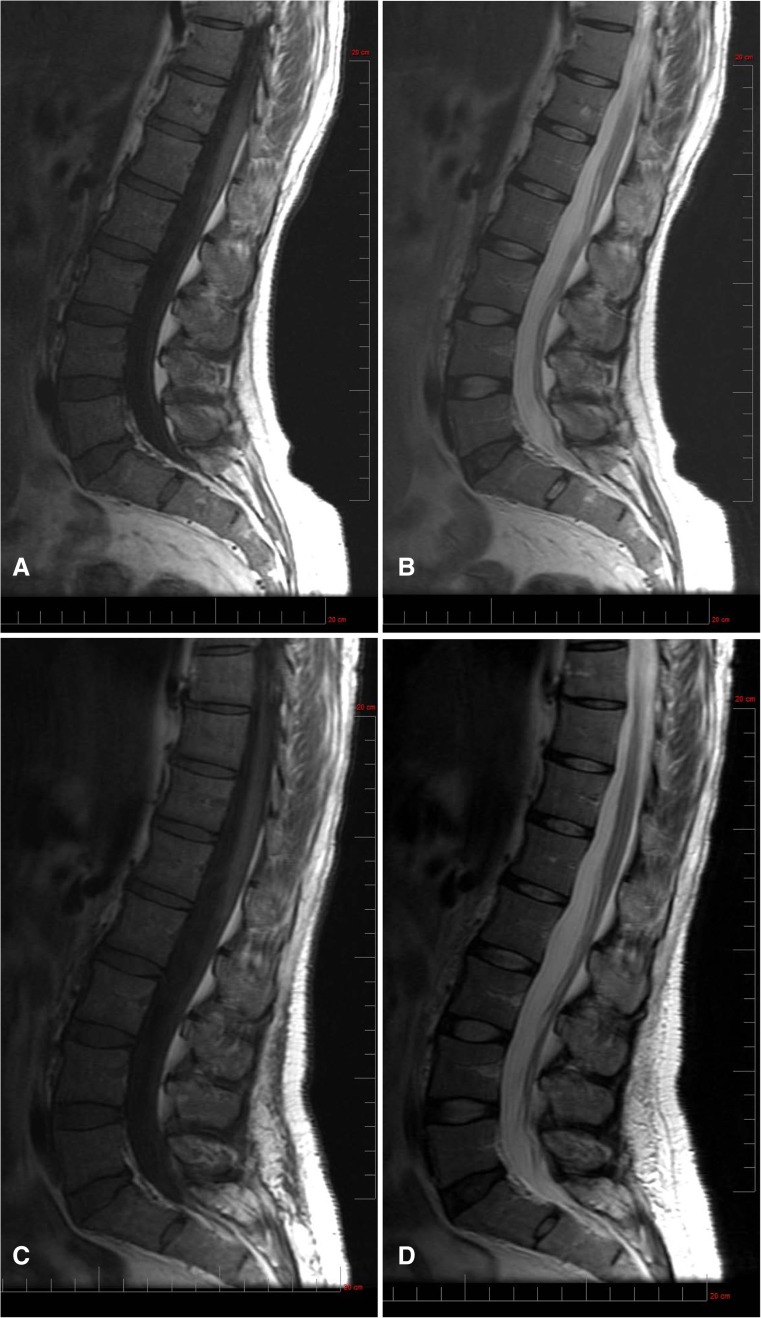
Assessment of olipudase alfa on bone marrow burden. Femur. Bone marrow burden changes in the coronal femur of patient 2, (female, 32 years old at baseline). Note the extent of hypointensity of the proximal epiphysis bone marrow at screening in the T1-weighted (a) and T2-weighted (b) images compared with the reduced amount and slightly hypointense diaphyseal bone marrow following 30 months of treatment (T1-weighted, c and T2-weighted, d). Full vertical scale bar, 20 cm. Spine. Bone marrow burden in the sagittal lumbar spine of patient 2. At screening, diffuse infiltration of the bone marrow is observed with T1-weighted isointensity of the non-diseased intervertebral discs (a) and T2-weighted hyperintense signal intensity of presacral fat (b). After 30 months of treatment, the infiltration of the bone marrow remains unchanged (T1-weighted, c) while the presacral fat is improved to slightly hyperintense (T2-weighted, d). Full vertical scale bar, 20 cm

Mean categorical scores (out of a possible total of 16) for BMB were similar at baseline (6.2 ± 2.5) and 30 months (5.6 ± 1.1). Patient 2 had the highest total BMB score of 10 at baseline, which improved by 3 points (to a score of 7) at 18 and 30 months. T1- and T2-weighted femur and spine images for patient 2 at baseline and after 30 months of olipudase alfa treatment are shown in Fig. 3. Hypointensity of the proximal epiphysis bone marrow observed at baseline was reduced following 30 months of treatment. In spine, diffuse infiltration of the bone marrow and hyperintense signal intensity of presacral fat observed at baseline was unchanged and improved, respectively, after 30 months of treatment.

### Patient reported outcomes

Mean BFI ± SD fatigue scores were 3.04 ± 2.29 at baseline and 2.44 ± 3.44 at 30 months. Mean BPI ± SD pain severity scores were 3.45 ± 2.77 at baseline and 2.90 ± 2.70 at 30 months, and mean BPI ± SD pain interference scores were 2.03 ± 1.58 at baseline and 3.29 ± 3.51 at 30 months. Most of the individual BFI and BPI pain severity scores were in the mild (0–3) or moderate (4–6) categories at all time points. Exceptions were patient 5, whose BPI pain was severe (7–10) at both baseline (6.8) and 30 months (7). BPI pain interference scores increased for patients 2 (2 at baseline, 8.1 at 30 months) and 3 (1.9 at baseline, 5.3 at 30 months). Fatigue reported by patient 2 was moderate (5.8) at baseline and severe (8.3) at 30 months.

## Discussion

Olipudase alfa is the first etiology-specific treatment in development for ASMD. This study demonstrates that treatment with olipudase alfa for 30 months is well-tolerated and associated with life-transforming sustained improvements in relevant disease clinical measures.

The 30 months safety profile was similar to the phase 1b study profile (Wasserstein et al 2015). There were no hypersensitivity reactions and no anti-drug antibodies were detected. No cytokine release syndrome has been observed in any patient exposed to olipudase alfa to date. Since IARs were not immunologic reactions, they are likely related to release of biologically active sphingomyelin metabolites, principally ceramide, which is a signaling intermediary in cytokine release, inflammation, and apoptosis (Spiegel et al 1996, Gulbins et al 2004). During the first 6 months of treatment, olipudase alfa doses elicited transient increases in plasma ceramide levels that generally peaked at 48 h post-infusion (Wasserstein et al 2015). Both pre- and post-infusion ceramide levels steadily decreased with each successive olipudase alfa infusion, plateauing after 3 months of treatment, and remaining stable through 30 months.

In this small cohort of patients, some patients had less severe disease at onset of ERT than others (for example, see data for patients 3 and 5 in Suppl. Table 1). Despite this heterogeneity, clinical improvements were observed for all patients, and benefits were sustained throughout 30 months. Even patients with the smallest organ volumes at baseline had clinically relevant reductions in liver and spleen volumes after treatment. Statistically significant improvements in liver and spleen volumes (mean percent decreases in liver volume of 31.2% and spleen of 39.3%) are comparable to responses of other lysosomal storage disorders to ERT. In Gaucher disease, the therapeutic goal for spleen volume is a 30 to 50% decrease during the first year of treatment, and for liver volume a 20 to 30% decrease within the first 2 years of treatment (Pastores et al 2004).

Patients with chronic visceral or chronic neurovisceral ASMD demonstrate worsening of infiltrative lung disease with age (Wasserstein et al 2004). Over 30 months of treatment there was a 35% increase from baseline in lung diffusing capacity, with prominent changes in the three patients with the lowest DL_CO_ at baseline. Improvements in lung disease scores observed during the first 6 months of treatment (Wasserstein et al 2015) continued during the subsequent 2 years of treatment, such that some parameters (e.g., GG appearance and RND) had normalized.

Atherogenic lipid profiles typically worsen with age in patients with chronic ASMD (Wasserstein et al 2004), and lipid abnormalities may be associated with early coronary artery disease (McGovern et al 2004). At baseline, patients were at mild-to-moderate risk for cardiovascular disease based on lipid profiles (Wasserstein et al 2015), and profiles improved over 30 months of treatment. Non-HDL cholesterol level is considered a good predictor of cardiovascular risk in many patient populations, and a desirable level is <3.37 mmol/L (< 130 mg/dL) (Jacobson et al 2015). All but one patient had non-HDL levels above 3.37 mmol/L before ERT, and at 30 months total cholesterol and HDL levels improved for all patients with non-HDL below the 3.37 mmol/L cutoff.

Skeletal complications are also prominent features of chronic ASMD with adult patients showing osteopenia or osteoporosis at one or more sites and decreased BMD compared to controls (Wasserstein and Godbold 2013). Improvements in BMD were noted in some patients in our study, particularly in the spine, suggesting that olipudase alfa may have a beneficial effect on BMD in adults with ASMD. In other lipid storage disorders with low BMD such as Gaucher disease, ERT in combination with antiresorptive therapy can improve osteopenia (Wenstrup et al 2004), although response of bone disease to ERT alone is slow in adult patients (Wenstrup et al 2007). However, bisphosphonates may not be appropriate in patients with ASMD due to inhibition of ASM activity (Arenz 2010). No study patient was receiving bisphosphonate therapy. While additional studies are necessary, results in this small population suggest that osteopenia may be improved with olipudase alfa alone.

Other clinical measures showed improvements or stability during ERT. Platelet counts and hemoglobin levels remained stable. Moderate levels of BMB were measured both at baseline and after 30 months of olipudase alfa treatment. In one patient with the greatest BMB, changes in bone marrow in the femur and spine were detectable after treatment, suggesting that BMB may be useful for semi-quantitative assessment of ERT in patients with more severe disease (Robertson 2007). Patients had mild to moderate levels of pain and fatigue at baseline that remained stable at 30 months for most patients. Worsening of patient reported outcomes for patient 3 was not associated with AEs. Patient 2 reported worsening of fatigue and pain with AEs characterized by flu-like symptoms after 1 year of ERT at the 3 mg/kg olipudase alfa dose. This patient has atypical lupus erythematosus and it is uncertain whether this contributed to fatigue and pain, AEs, and inflammatory cytokine fluctuations. Decreasing olipudase alfa to 1 mg/kg/week in this patient has had no impact on AE incidence, fatigue or pain. The constellation of AEs that triggered the dose reduction has not changed, nor did the patient miss any ERT infusions due to intolerance. At the lower olipudase alfa dose (12 months of exposure), the patient continues to have clinical benefit including reduced spleen and liver volumes as well as improvement in percent predicted DLco, sustained clearance of infiltrative HRCT parameters, and stabilization of biomarkers.

Chitotriosidase, a well-known biomarker for therapeutic monitoring during ERT in Gaucher disease (Guo et al 1995) and a marker of chronic inflammatory diseases (Boot et al 2010), steadily decreased during olipudase alfa treatment. Lyso-SPM, the deacylated form of sphingomyelin, decreased in DBS, suggesting utility as a biomarker for monitoring ERT outcomes reflected by steady decreases as patients undergo debulking of sphingomyelin, followed by stability during long-term treatment. Lyso-SPM is elevated approximately fivefold in DBS from patients with chronic visceral ASMD (Chuang et al 2014). While lyso-SPM in DBS was the protocol-specified assessment for this clinical trial, in clinical practice monitoring of plasma lyso-SPM levels may be accomplished using recently developed methods (Kuchar et al 2017); in ongoing pediatric and confirmatory adult clinical trials with olipudase alfa, plasma lyso-SPM is the protocol-designated assessment.

Inherent limitations include the small number of study patients; however, the longitudinal data set is robust. The efficacy and safety of olipudase alfa in adults with ASMD are being confirmed in a phase 2/3 trial, ASCEND (NCT02004691; EudraCT:2015–000371-26), and are also under investigation in pediatric patients (NCT02292654; EudraCT:2014–003198-40). In summary, this open-label extension study of olipudase alfa demonstrates that treatment with olipudase alfa for 30 months is well-tolerated and clinically effective.

## Electronic supplementary material

Suppl. Table 1 Patient demographics and baseline characteristics (Wasserstein et al 2015) (DOCX 18 kb)

Suppl. Fig. 1 Fasting lipid parameters at baseline and during treatment (30 months) with olipudase alfa. Mean (SD) pre-infusion fasting levels of total cholesterol (a), triglycerides (b), HDL cholesterol (c), and LDL cholesterol (d) (PDF 1361 kb)

## Abbreviations

ALT: Alanine aminotransferase
ASM: Acid sphingomyelinase
ASMD: Acid sphingomyelinase deficiency
AE: Adverse event
AST: Aspartate aminotransferase
BMB: Bone marrow burden
BMD: Bone mineral density
BFI: Brief Fatigue Inventory
BPI-SF: Brief Pain Inventory-Short Form
DLco: Diffusing capacity of the lung for carbon monoxide
DBS: Dried blood spot
DXA: Dual-energy X-ray absorptiometry
ERT: Enzyme replacement therapy
GGT: Gamma glutamyl transferase
GG: Ground glass appearance
HDL-C: High density lipoprotein
HRCT: High-resolution computed tomography
ILD: Interstitial lung disease
IARs: Infusion associated reactions
LC/MS/MS: Liquid chromatography-tandem mass spectrometry
lyso-SPM: lyso-sphingomyelin
LTS: Long-term study
LDL-C: Low density lipoprotein
MN: Multiples of normal
NPD: Niemann-Pick disease
RD: Reticulonodular density
TC: Total cholesterol
