# Supplementary material for: Olipudase alfa for treatment of acid sphingomyelinase deficiency (ASMD): safety and efficacy in adults treated for 30 months
Source: J Inherit Metab Dis. 2018 Jan 5;41(5):829–38. doi: 10.1007/s10545-017-0123-6 (PMC6133173; doi:10.1007/s10545-017-0123-6)
Supplement: Supplementary file 1 — Patient demographics and baseline characteristics (Wasserstein et al 2015) (DOCX 18 kb) [file 10545_2017_123_MOESM1_ESM.docx]

**Supplemental Table 1: Patient Demographics and Baseline Characteristics (Wasserstein et al. 2015)**

|  | **Patient ID** | | | | |  |
| --- | --- | --- | --- | --- | --- | --- |
|  | **1** | **2** | **3** | **4** | **5** |  |
|  | Male | Female | Female | Male | Male | **Mean (SD)** |
| **ASMD Symptom onset age (years)** | 2 | 1 | 6 | 0 | 12 | 4.2 (4.9) |
| **ASMD Diagnosis age (years)** | 2 | 2 | 12 | 8 | 12 | 7.2 (5.0) |
| **Age at first olipudase alfa infusion (years)** | 31 | 32 | 47 | 28 | 22 | 32.6 (9.4) |
| **Spleen Volume (MN)^a^** | 14.49 | 17.92 | 7.41 | 16.07 | 7.96 | 12.77 (4.81) |
| **Liver Volume (MN)^a^** | 2.23 | 2.20 | 1.21 | 1.76 | 1.29 | 1.74 (0.48) |
| **DL_CO_ (% predicted)^b^** | 43.7 | 48.0 | 77.0 | 43.0 | 80.0 | 58.3(18.5) |
| **TC (mmol/L)^c^** | 4.70 | 3.83 | 5.26 | 4.66 | 3.63 | 4.42 (0.67) |
| **HDL-C (mmol/L)^d^** | 0.32 | 0.36 | 0.96 | 0.31 | 0.57 | 0.50 (0.28) |
| ***Non-HDL (mmol/L)^e^*** | *4.38* | *3.47* | *4.30* | *4.35* | *3.06* | *3.91 (0.61)* |
| **LDL-C (mmol/L)^f^** | 3.38 | 2.59 | 3.32 | 2.69 | 2.25 | 2.85 (0.49) |
| **VLDL-C (mmol/L)^g^** | 0.88 | 0.88 | 0.98 | 1.66 | 0.80 | 1.04 (0.35) |
| **Triglycerides (mmol/L)^h^** | 2.20 | 1.55 | 1.14 | 4.35 | 1.38 | 2.12 (1.31) |

ASM = acid sphingomyelinase; ASMD = acid sphingomyelinase deficiency; C = cholesterol; DL_CO_=lung diffusion of carbon monoxide; HDL=high-density lipoprotein; LDL=low-density lipoprotein; MN=multiples of normal; SD=standard deviation; TC =total cholesterol; VLDL=very low-density lipoprotein.

^a^ MN, multiples of normal calculated assuming normal spleen volume (L) is 0.2% body weight (kg), and normal liver volume (L) is 2.5% body weight (kg)

^b^ Normal DLco >80%; Mildly reduced >60% to ≤ 80%; Moderately reduced 40-60%; Severely reduced < 40%

^c^ Total cholesterol normal range: US <5.18 mmol/L; UK 0-3.9 mmol/L

^d^ HDL normal range: US male >0.777; US female >0.9065 mmol/L; UK >1.2 mmol/L

^e^ calculated as the difference between TC and HDL-C (Jacobson et al. 2015)

^f^ LDL normal range: US <3.3411 mmol/L; UK 0-2 mmol/L

^g^ VLDL normal range: US <0.518 mmol/L; UK 0.09-0.71 mmol/L

^h^ Triglycerides normal range: <1.7 mmol/L
