# Supplementary material for: Olipudase alfa for treatment of acid sphingomyelinase deficiency (ASMD): safety and efficacy in adults treated for 30 months
Source: J Inherit Metab Dis. 2018 Jan 5;41(5):829–38. doi: 10.1007/s10545-017-0123-6 (PMC6133173; doi:10.1007/s10545-017-0123-6)
Supplement: Supplementary file 2 — Fasting lipid parameters at baseline and during treatment (30 months) with olipudase alfa. Mean (SD) pre-infusion fasting levels of total cholesterol (a), triglycerides (b), HDL cholesterol (c), and LDL cholesterol (d) (PDF 1361 kb) [file 10545_2017_123_MOESM2_ESM.pdf]

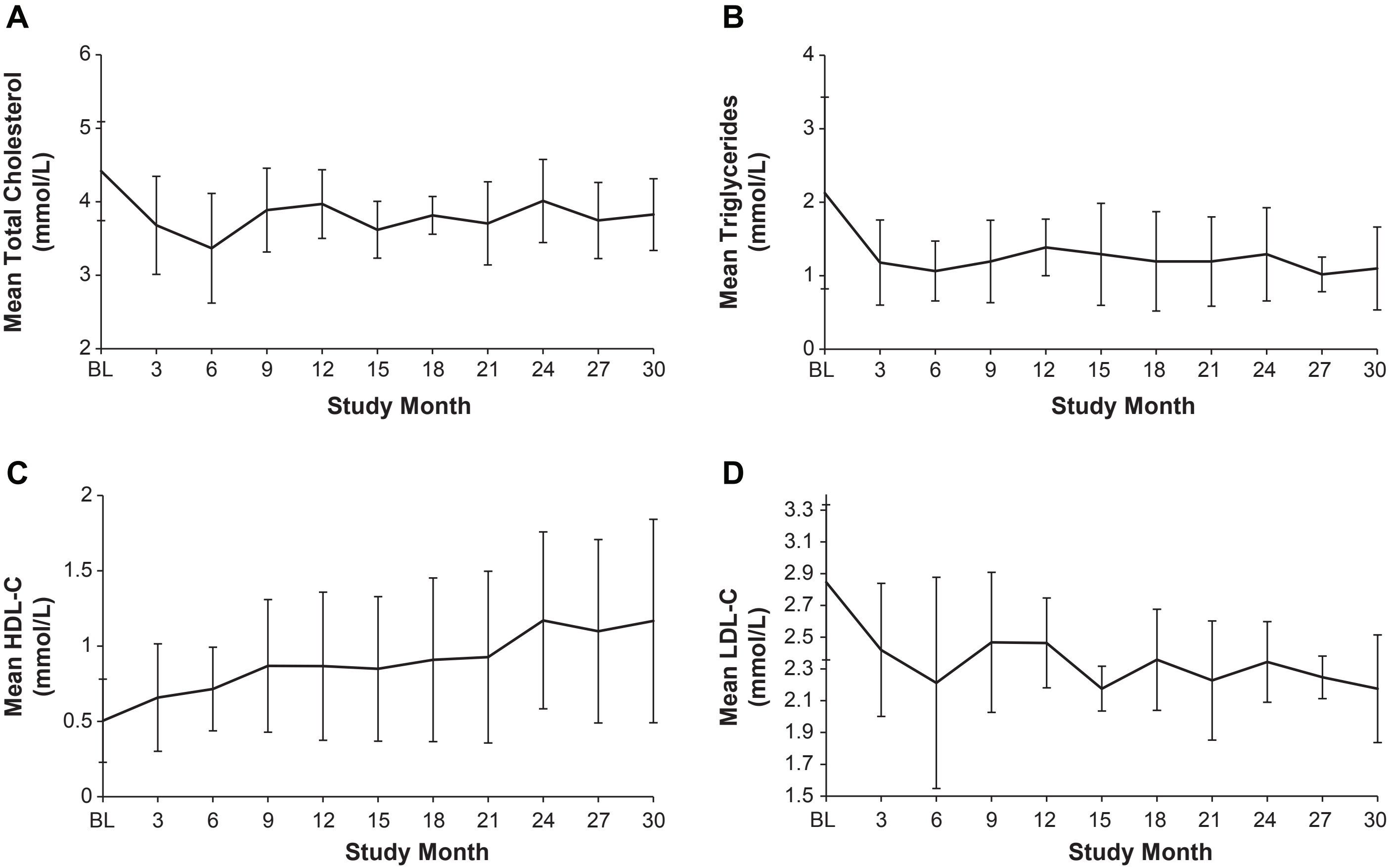

Total cholesterol normal range: US <5.18 mmol/L; UK 0-3.9 mmol/L
HDL-C normal range: US male >0.777; US female >0.9065 mmol/L; UK >1.2 mmol/L
LDL-C normal range: US <3.3411 mmol/L; UK 0-2 mmol/L
Triglycerides normal range: <1.7 mmol/L
